# Multifunctional central pattern generator controlling walking and paw shaking

**DOI:** 10.1186/1471-2202-15-S1-P181

**Published:** 2014-07-21

**Authors:** Brian Bondy, Alexander Klishko, Boris Prilutsky, Gennady Cymbalyuk

**Affiliations:** 1 Neuroscience Institute, Georgia State University, Atlanta, Georgia, 30302, USA; 2 School of Applied Physiology, Georgia Institute of Technology, Atlanta, Georgia, 30332, USA

## 

**Figure 1:**
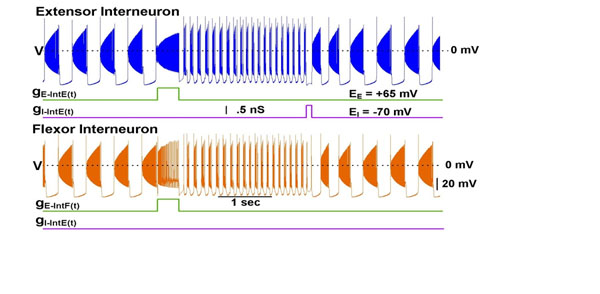
Two mutually inhibitory interneurons, IntE (Extensor Interneuron) and IntF (Flexor Interneuron) produce alternating bursting activity at approximately 1.6 Hz representing walking pattern. A switch to paw shaking is executed by a pulse of excitatory conductance delivered to both cells for 1 second. The paw shake rhythm is represented by a 9 Hz bursting regime. An inhibitory conductance activated for .1 second in IntF causes a large rebound burst and a fast transition back to the walking rhythm.

Central pattern generators (CPGs) are oscillatory neuronal networks controlling rhythmic motor tasks such as breathing and walking. A multifunctional CPG can produce multiple patterns, e.g. patterns with different periods [1-5]. Here, we investigate whether a pair of cat behaviors -- walking and paw shaking -- could be controlled by a single multifunctional CPG exhibiting multistability of oscillatory regimes. In experiments, both behaviors can be elicited in a spinalized cat, and there is evidence that the same circuitry is used for both rhythms [2,3]. We present a parsimonious model of a half-center oscillator composed of two mutually inhibitory neurons. These cells contains two slowly inactivating inward currents, a persistent Na^+^ current (I_NaP_) and a low voltage activated Ca^++^ current (I_CaLVA_). The dynamics of the multifunctional CPG is based on that the I_CaLVA_ inactivates much slower than I_NaP_ and at the more hyperpolarized membrane potentials. Here, we demonstrate the co-existence of two rhythms (Figure 1). At first, the model demonstrates walking pattern. A switch from a slow, 1-2 Hz walking rhythm to fast, 7-10 Hz paw shake rhythm was elicited by a pulse of conductance of excitatory current delivered to extensor and flexor neurons. Then, a switch back to walking was triggered by a shorter pulse of conductance of inhibitory current delivered to the extensor neuron.

The CPG model was also incorporated into a neuromechanical model of a cat hindlimb in the AnimatLab environment [6]. The model provides a cellular mechanism of multifunctional CPG operation.
